# Unusual Structures Are Present in DNA Fragments Containing Super-Long Huntingtin CAG Repeats

**DOI:** 10.1371/journal.pone.0017119

**Published:** 2011-02-11

**Authors:** Daniel Duzdevich, Jinliang Li, Jhoon Whang, Hirohide Takahashi, Kunio Takeyasu, David T. F. Dryden, A. Jennifer Morton, J. Michael Edwardson

**Affiliations:** 1 Department of Pharmacology, University of Cambridge, Cambridge, United Kingdom; 2 Laragen, Inc., Culver City, California, United States of America; 3 Laboratory of Plasma Membrane and Nuclear Signaling, Graduate School of Biostudies, Kyoto University, Yoshida Konoe-cho, Sakyo-ku, Kyoto, Japan; 4 EaStCHEM School of Chemistry, University of Edinburgh, The King's Buildings, Edinburgh, United Kingdom; Charité-Universitätsmedizin Berlin, Germany

## Abstract

**Background:**

In the R6/2 mouse model of Huntington's disease (HD), expansion of the CAG trinucleotide repeat length beyond about 300 repeats induces a novel phenotype associated with a reduction in transcription of the transgene.

**Methodology/Principal Findings:**

We analysed the structure of polymerase chain reaction (PCR)-generated DNA containing up to 585 CAG repeats using atomic force microscopy (AFM). As the number of CAG repeats increased, an increasing proportion of the DNA molecules exhibited unusual structural features, including convolutions and multiple protrusions. At least some of these features are hairpin loops, as judged by cross-sectional analysis and sensitivity to cleavage by mung bean nuclease. Single-molecule force measurements showed that the convoluted DNA was very resistant to untangling. *In vitro* replication by PCR was markedly reduced, and TseI restriction enzyme digestion was also hindered by the abnormal DNA structures. However, significantly, the DNA gained sensitivity to cleavage by the Type III restriction-modification enzyme, EcoP15I.

**Conclusions/Significance:**

“Super-long” CAG repeats are found in a number of neurological diseases and may also appear through CAG repeat instability. We suggest that unusual DNA structures associated with super-long CAG repeats decrease transcriptional efficiency *in vitro*. We also raise the possibility that if these structures occur *in vivo*, they may play a role in the aetiology of CAG repeat diseases such as HD.

## Introduction

At least 30 genetic neurodegenerative diseases involve the expansion of specific DNA triplet repeat sequences [1], [2]. These include Fragile X syndrome (CGG repeat), Friedreich's ataxia (GAA repeat), myotonic dystrophy type I (CTG repeat), and Huntington's disease (HD; CAG repeat). Depending on the disease, the repeat sequence can be located at various positions relative to the coding region of the gene. Hence, the role of the repeat in the aetiology of the disease will vary. For instance, the CGG repeat diseases appear to involve methylation of the DNA containing the repeats, leading to gene silencing. In contrast, the presence of an expanded CAG repeat in the coding region of *HTT*, the gene encoding Huntingtin (htt) leads to the generation of an expanded polyglutamine tract in the expressed protein, which produces neurotoxic effects through a gain of function [1]. As the HD-associated CAG repeat expansion is exon-localized, its possible role in controlling gene expression has been largely overlooked.

The threshold CAG repeat length that causes HD is around 36, and there is an approximate correlation between the age-at-onset of the disease and the length of the CAG tract [3], [4], [5]. Patients with 40–42 repeats tend to get the disease in mid-life (35–40 years), whereas patients with more than 60 repeats are afflicted as juveniles [1]. One factor that might blur the relationship between age-at-onset and repeat length is instability of the repeat length, which is known to occur both in humans [6], [7], [8], [9], [10] and in mouse models of HD [11], [12], [13], [14], [15]. Indeed, ‘super-long’ expansions of CAG repeats up to 1000 have been found in neurons from the brains of patients with adult-onset HD, despite much shorter (41–51) inherited CAG repeats being measured in blood analyses [9], [13].

To assess the functional significance of such super-long repeats, R6/2 mice carrying the first exon of the human HD gene with a pathological CAG repeat expansion [16], have been bred with repeat sizes up to around 500 CAG repeats [17]. Surprisingly, it was found that mice carrying the super-long repeat had a delayed onset of the disease phenotype and prolonged survival. The survival data showed a pronounced turning point at around 300 CAG repeats, indicating a fundamental change in phenotype. A concurrent study found a similar turning point [18]. Both studies showed a shift from predominantly intranuclear to predominantly cytoplasmic inclusions, suggesting that above a certain length the mutant protein cannot access the nucleus. Furthermore, the latter study reported that in mice with more than about 335 CAG repeats there was a >60% reduction in both mRNA levels for the transgene and expression of mutant protein compared with mice with 150 CAG repeats, suggesting that transcription of the gene was being inhibited. Significantly, a previous study has shown that HD homologue (Hdh) mRNA levels in knock-in mice containing 150 CAG repeats were already reduced compared with wild type, suggesting that a reduction in transcription becomes apparent even below the 300 CAG ‘turning point’ [19]. Nevertheless, it is possible that both a reduction in mRNA expression and nuclear entry of the mutant protein could contribute to the delayed phenotype in mice with longer repeats.

In this study we set out to test the hypothesis that the observed change in phenotype of the R6/2 mouse involves the generation of unusual, non-B-form DNA at super-long CAG repeat lengths. There is already considerable evidence that the various triplet repeats generate unusual DNA structures, such as triplexes, hairpins forming in one or both strands of the DNA, slipped-strand DNA, and G-quadruplexes [20]. These structures have been detected through their effects on the mobilities of DNA on agarose gels (e.g. [21]), and have also been directly observed by electron microscopy (EM; [22], [23]). Significantly, EM analysis requires extensive sample preparation and the imaging of the sample *in vacuo*, processes that result in the loss of some structural information. In contrast, atomic force microscopy (AFM) is able to image DNA at the single molecule level with only minimal sample preparation [24], [25], revealing features that are not detectable using EM imaging. For instance, we have previously used AFM to visualize G-quadruplexes in DNA containing a G-rich fragment of the mouse Sγ3 immunoglobulin switch region [26]. We found that the G-loops are asymmetric, and we showed directly that the G-rich strand forms a condensed structure, with a height appropriate to a four-stranded structure. This is in stark contrast to the extended single-stranded regions seen in the same DNA by EM [27], which are not consistent with the presence of G-quadruplexes in the form visualized. AFM images of triplet repeat DNA have been published before [28]; however, to the best of our knowledge, a systematic analysis of the structural features appearing at different repeat lengths has not been undertaken, particularly at super-long repeat lengths. Here we have used AFM to image DNA generated by PCR using genomic DNA isolated from tail snips taken from R6/2 mice as a template. We show that PCR-generated DNA with super-long repeats contains a variety of unusual structures, including hairpin loops and loop-mediated gross malformations, which, if they exist *in vivo*, would likely contribute to abnormal transcriptional regulation of the gene.

## Materials and Methods

### Generation of DNA

DNA samples containing various CAG repeat lengths were generated by PCR from tail snips of either wild type or R6/2 mice [16], which carry the first exon of the human HD gene with a pathologically expanded repeat. The repeat length in the R6/2 mice was originally around 140–150 CAG, but cohorts of mice have been generated with repeat lengths up to 485 [17]. Mouse husbandry was as described previously [29]. In some experiments, the forward and reverse PCR primers were tagged with biotin and DIG, respectively. PCR was carried out essentially as described in [11] with minor modifications. 35 cycles of PCR were carried out, using Taq polymerase (Applied Biosystems), with an annealing temperature of 65°C. The resulting PCR products from the R6/2 mice have 54 bp (5′) and 77 bp (3′) sequences flanking the CAG repeat core. Note that the GeneScan profiles of the PCR products revealed a range of sizes, and the peak sizes were used in our analyses. The quoted repeat lengths are therefore not absolute, and likely carry an error of 1–2%. Note also that the CAG repeat number measured by GeneMapper differs from that measured by sequencing. To convert the CAG repeat numbers determined by the GeneMapper technique to the CAG repeat number determined by sequencing technique (which more closely represents the true CAG repeat number in both HD transgenic mice and HD patients) the following formula was applied (Li and Whang, unpublished): True CAG repeat number  = 1.0425 x GeneMapper CAG repeat number +1.2088.

Where appropriate, DNA was purified using a DNA purification and concentrator kit (Zymo Research).

Single-stranded (ss) DNA was PCR amplified from the human σ-1 receptor gene in the pGH19 plasmid, kindly provided by Prof. M.B. Jackson (Department of Physiology, University of Wisconsin, Madison, USA). Primers were: forward, 5′-TCCAAGCTTGATGCAGTGGGCCGTGGGC-3′; reverse, 5′-TAAGCGGCCGCAGGGTCCTGGCCAAAGAG-3′. A 50-fold excess of forward primer was used, with 35 PCR cycles at an annealing temperature of 45°C, using Pfu Turbo DNA polymerase (Stratagene). Samples were purified using a QIAquick gel extraction kit.

### Enzyme Digestion

Extensive digestion by the heat-resistant restriction enzyme TseI (New England BioLabs) was carried out for 60 min at 80°C in buffer containing 50 mM potassium acetate, 20 mM Tris-acetate, pH 7.9, 10 mM magnesium acetate and 1 mM dithiothreitol. The reaction was stopped by addition of 10% glycerol and 10 mM EDTA (final concentrations). Limited digestion was carried out at room temperature for 10 min, with one-fifth of the amount of enzyme used in the full digestion.

Mung bean nuclease (New England BioLabs) digestion was carried out for 30 min at 30°C in buffer containing 50 mM sodium acetate, pH 5.0, 30 mM NaCl and 1 mM ZnSO_4_. Where appropriate, nuclease-treated DNA was melted by incubation at 94°C for 5 min. In this case, the nuclease was first inactivated by addition of 10% glycerol and 10 mM EDTA.

EcoP15I (New England Biolabs) digestion was carried out for 1 h at 37°C with 0.03 U/µl of enzyme in buffer containing 100 mM NaCl, 50 mM Tris-HCl, pH 7.9, 10 mM MgCl_2_, 1 mM dithiothreitol and 1 mM ATP. At the end of the digestion the enzyme was inactivated by a 20-min incubation at 65°C.

In all cases, DNA was purified using a QIAquick gel extraction kit prior to AFM imaging.

### Sample Preparation

Disks of ruby muscovite mica (Agar Scientific) were attached to 13-mm steel pucks (Agar Scientific) using Loctite super-glue (Henkel Loctite Ltd.). The mica was cleaved with Scotch tape immediately prior to sample deposition to reveal an atomically flat surface. DNA was prepared at a concentration of ∼0.04 ng/µl in imaging buffer (10 mM MgCl_2_, 10 mM Tris-Cl, pH 8.0). Droplets (45 µl) were applied to the freshly-cleaved mica, and incubated at room temperature for 5–10 min. The surface was washed ten times with 1 ml biotechnology performance certified (BPC) water (Sigma-Aldrich). Excess liquid was removed using a gentle stream of water-filtered nitrogen, and samples were placed in a non-vacuum desiccator overnight. Where appropriate, mica was treated with 50 µl of 0.1% poly-L-lysine (Sigma-Aldrich) for 10 min followed by washing, before adsorption of DNA.

### AFM Imaging

Imaging was performed using a Multimode Nanoscope IIIa atomic force microscope (Veeco Digital Instruments, Santa Barbara, CA, U.S.A.) fitted with an AS130 ‘J’ scanner. All samples were imaged in air using tapping mode. Micro Cantilever silicon tips with a spring constant of ∼42 N/m (Olympus) driven at a resonant frequency of ∼300 kHz were used as probes. The instrument was operated with Veeco's Nanoscope software (v5.31r1) and Nanoscope IIIa interface (Digital Instruments). Integral gain was set to 0.2, proportional gain to 0.4, scan rate to 5 Hz, and z-limit to 1 µm. Images contained 512×512 pixels of height data, and image area was set between 1 µm and 4 µm, depending on DNA size and the density of particles on the surface. Colour was used to encode height. Scales were adjusted between experiments to present clear images of the DNA. These adjustments were necessary because of the variation in both tip performance and background quality between experiments.

### Image Analysis

First order ‘flattening’ was applied to raw images in Nanoscope to compensate for the intrinsic bow resulting from the nature of the AFM's piezo-actuated scanner. The height scale, colour contrast, and colour offset were set individually for each image to maximize clarity. (These settings do not alter fundamental image values). Particle volumes were assayed with the ‘grain analysis’ function in SPIP (Image Metrology). DNA length was measured using the ‘line plug-in’ running in ImageJ (1.37v, NIH).

### Force Measurement

All procedures were carried out at room temperature. AFM cantilevers made of silicon nitride with a specified spring constant of 0.02 N/m (OMCL-TR400PSA; Olympus Co. Ltd., Japan), and freshly-cleaved mica sheets, were treated with 3-aminopropyltriethoxysilane (APTES; Sigma) and *N*,*N*-diisopropylethylamine for 1 h. After washing with MilliQ water, cantilevers and mica were incubated with 1 mM glutaraldehyde for 10 min. After further washing in water, the cantilevers were dipped into streptavidin (0.1 mg/ml) in phosphate-buffered saline (PBS), and incubated for 20–30 min. The mica was treated with anti-DIG antibody (10 µg/ml) for 20–30 min. Cantilevers and mica were then washed with PBS, and with 50 mM KCl, 10 mM Tris-HCl buffer, pH 7.6. The cantilevers were dipped into the appropriate DNA solution (3–5 ng/ml) for 10 min. After washing in Tris/KCl buffer, cantilevers and mica were used immediately.

Force measurements were performed with a Digital Instruments Multimode atomic force microscope equipped with an E-scanner and a Nanoscope IIIa controller with an in-line electronics extender module (Veeco Digital Instruments, Santa Barbara, CA, U.S.A.). Measurements were carried out in Tris/KCl buffer (above). The pulling speed was 100 nm/s, the pulling distance was 500 nm, and the trigger point was set at 50 nm. The data were analysed using SPIP. Rupture forces were obtained from force-extension curves.

## Results

### Apparent Anomalies in Genotyping of Double Mutant Mice with Super-Long CAG Repeats

**Figure 1:**
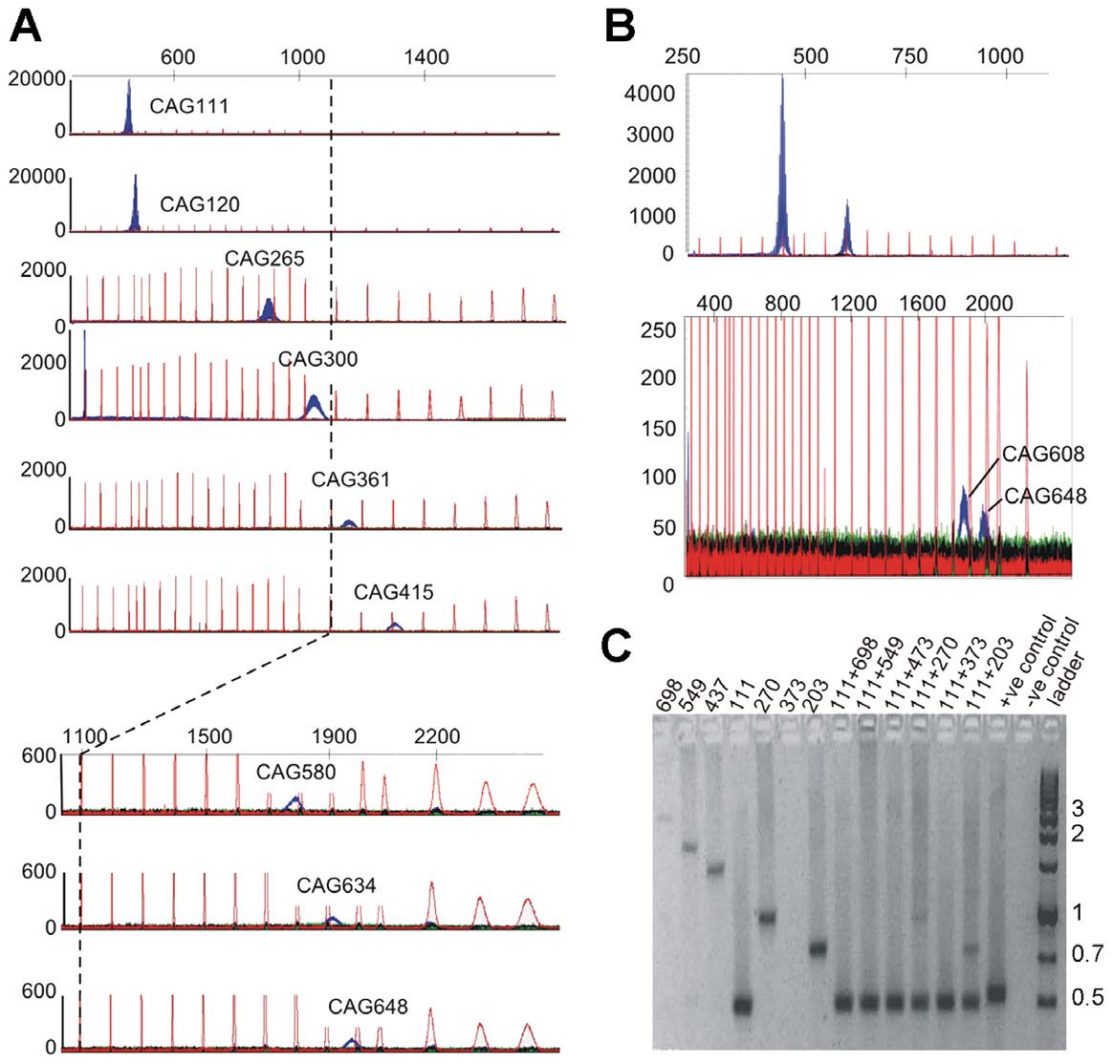
CAG repeat length affects PCR efficiency and accuracy. (A) GeneScan profiles of PCR products containing various CAG repeat lengths, showing that peak heights fall and peak widths rise as CAG repeat length rises. (Note the changing values on the y-axes). The red peaks indicate the positions of size markers (bp). A bp-scale is shown at the top. (B) GeneScan profiles of PCR products from two double mutant mice, showing that in both cases the longer repeat is under-represented. (C) Agarose gel of PCR products containing various repeat lengths. CAG repeat lengths are indicated above each lane. The left-hand side shows PCR products generated from DNA taken from mice carrying single copy of the transgene of varying sizes; the right-hand side shows PCR products from reactions containing equal amounts of the DNA from two of the mice shown on the left hand side; one carrying a CAG repeat of 111 and one other carrying a longer repeat. Note that the PCR reaction in the lane labelled 373 has failed.

This study was prompted by anomalies encountered in the routine genotyping of R6/2 mice by PCR, using genomic DNA isolated from tail snips as a template. It was found that as the CAG repeat length increased, PCR yields fell, and the PCR products became progressively less well defined, a finding that has been reported previously by others [30], [31], [32]. This phenomenon can be seen in GeneScan profiles (Figure 1A). This became problematic when genotyping mice with CAG repeats longer than 400, and even more so when we tried to measure the CAG repeat lengths from double mutant mice. Because mice with super-long CAG repeats are fertile, offspring carrying two copies of the transgene can be generated. However, matings between hemizygotic mice appeared to generate significantly fewer double mutant offspring than expected when the repeat length of the parents was long (350–500 CAG repeats), but not when it was short (250–260 CAG repeats). On those occasions when two peaks were seen, GeneScan profiles typically showed that the longer of the two repeats was under-represented and more diffuse (Figure 1B). More commonly, there was no second peak, although subsequent phenotyping and histological analysis [17] showed that the proportion of mice carrying two copies of the transgene was 0.25, as expected. The unequal amplification of differently sized alleles was recapitulated *in vitro* (Figure 1C). When PCR reactions were run using template DNA from mice carrying a single copy of the transgene (lanes 1–6 on the left hand side of the gel), PCR products became increasingly faint as the repeat length increased. The preferential PCR amplification of shorter template was shown in an experiment in which template was generated by *in vitro* mixing DNA from two different animals. When the template DNA from a mouse carrying a CAG repeat of 111 was mixed with that of a mouse carrying a longer CAG repeat (Figure 1C; right-hand side of the gel), the longer of the two PCR products was clearly under-represented or, with the longest repeat sizes, undetectable. This phenomenon was reported some time ago for CAG repeats in the human androgen receptor gene [33] and for GAA repeats in the Friedreich's ataxia gene [34]. Interestingly, the former paper contained speculation that the HD locus might show the same effect, as we have now demonstrated.

### AFM Imaging Reveals Unusual Structures in DNA with Super-Long CAG Repeats

**Figure 2:**
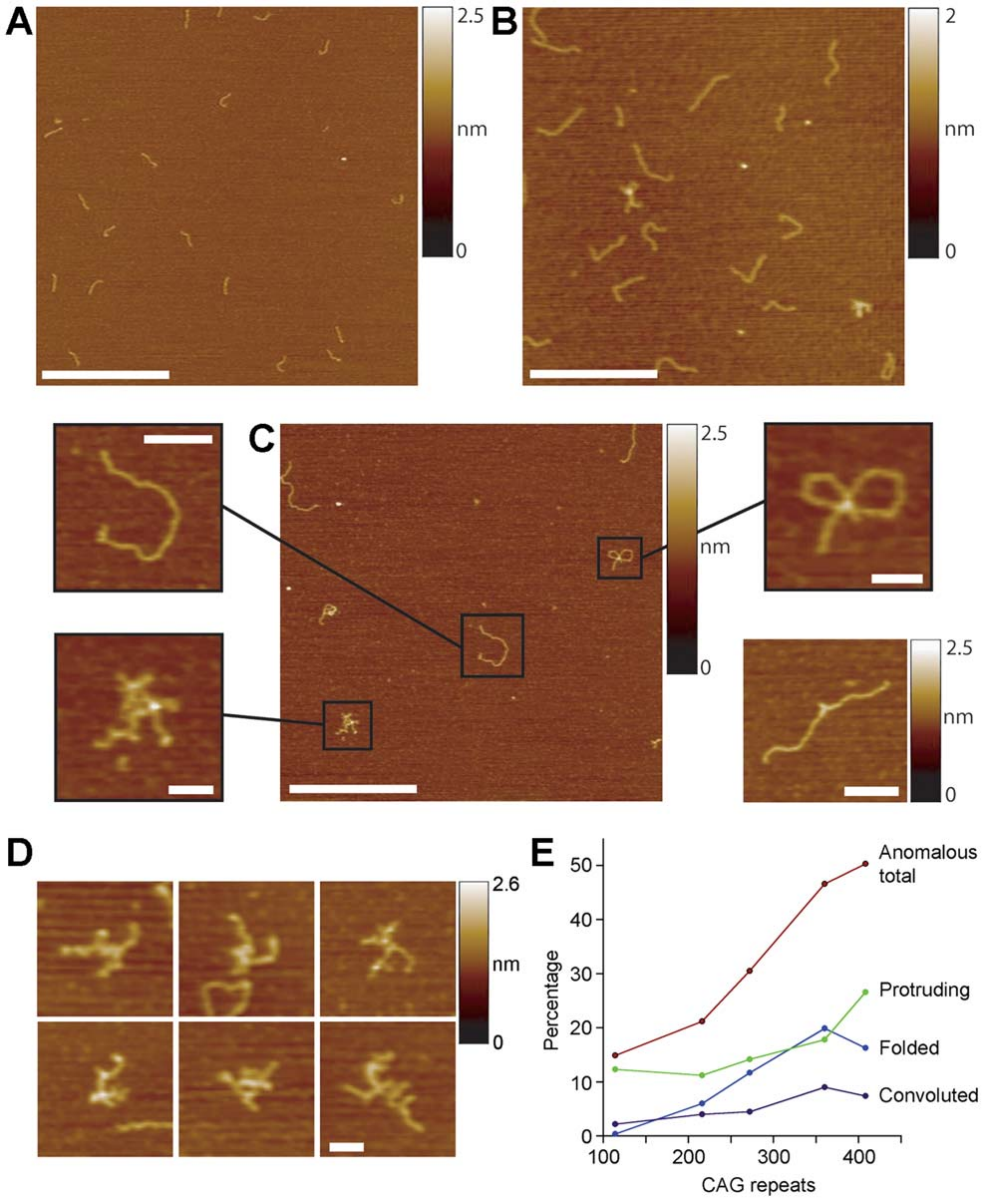
Unusual structures occur in super-long CAG repeat DNA. (A) AFM image of wild type DNA with 8 CAG repeats (total length 298 bp). Scale bar, 500 nm. A colour-height scale is shown at the right. (B) DNA with 216 repeats (total length 779 bp). Scale bar, 500 nm. (C) DNA with 360 CAG repeats (total length 1211 bp). Scale bar, 500 nm. DNA fragments were classified into ‘normal and linear’ (inset, upper left; scale bar 100 nm), ‘convoluted’ (lower left; scale bar, 50 nm), ‘folded’ (upper right; scale bar, 50 nm), or ‘protruding’ (lower right, derived from another image of the same PCR product; scale bar 100 nm). (D) Gallery of zoomed images showing examples of convoluted DNA. (E) Graph showing the relationship between the percentage of DNA molecules with unusual structures and CAG repeat length. Numbers of DNA molecules analysed ranged from 1163 (114 repeats) to 203 (408 repeats).

**Figure 3:**
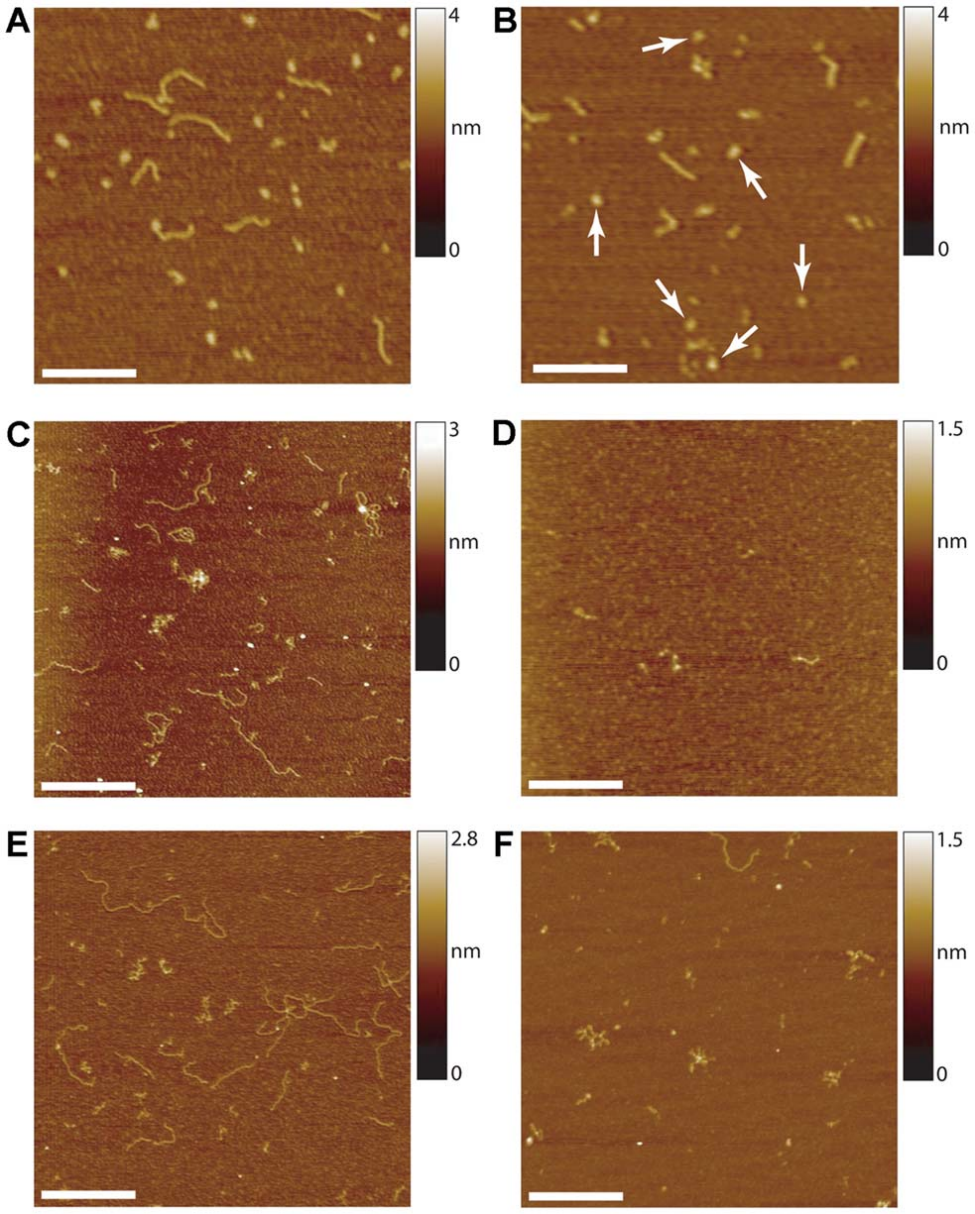
Convoluted DNA does not contain extensive regions of ssDNA. (A) AFM image of human σ-1 receptor DNA, generated by PCR using a 50-fold excess of forward primer. Two types of structure can be seen: 690-bp dsDNA, which is linear and ‘normal’, and ssDNA, which is very dense and compacted. Scale bar, 250 nm. (B) σ-1 receptor DNA after extensive Tse1 digestion at 80°C. The enzyme has cleaved the dsDNA, leaving shorter fragments, but the ssDNA (arrows) remains unaffected. Scale bar, 250 nm. (C) DNA containing 585 CAG repeats. Note that some molecules are linear, whereas others are folded or convoluted. Scale bar, 500 nm. (D) 585-repeat DNA after extensive Tse1 digestion at 80°C. A dense homogenous ‘background’ of short (∼6-bp) fragments remains, together with a few aggregations of these fragments, and one fragment of ∼60 bp, which likely represents a flanking region. Scale bar, 250 nm. (E) Undigested 585-repeat DNA. Scale bar, 500 nm. (F) 560-repeat DNA after limited Tse1 digestion at room temperature. The enzyme has preferentially cleaved linear regions of DNA, leaving behind the anomalous regions. Scale bar, 250 nm.

A potential explanation of the results described above is that super-long DNA adopts unusual structures that reduce the efficiency of the PCR reaction. Such a phenomenon occurring *in vivo* might also account for the observed reduction in transcription of the gene encoding htt as repeat length entered the super-long range [18]. To test this idea, we analysed DNA samples of various CAG repeat lengths by AFM imaging. We found that the structural profile of the DNA changed significantly as repeat length increased (Figure 2). DNA generated from wild type mice, appeared as short linear molecules of length 96±5 nm (S.D.; n = 100; Figure 2A). The PCR fragment generated from the region containing the (CAG)_2_CAA(CAG)_4_ sequence carried by wild type mice was expected to have a total length of 298 bp. We have shown in a previous AFM-based study of plasmid DNA [25] that an appropriate nm-to-bp conversion factor is 0.334 nm/bp. 298-bp molecules should therefore be 99 nm long, very close to the observed value. Note also that we have previously used AFM to image various DNA molecules that do not contain CAG repeats and have only ever seen linear structures (e.g. [24], [25]). DNA with 216 CAG repeats (total length 779 bp, including flanking sequences) also appeared predominantly ‘normal’ and linear, with an average length of 240±14 nm (n = 100), close to the expected 260 nm (Figure 2B). In contrast, when the DNA contained 360 CAG repeats (total length 1211 bp), various DNA structures became apparent (Figure 2C; see also Figure 3C for an image of 585-repeat DNA). Specifically, some of the DNA still appeared normal and linear (inset, upper left), but unusual structures were also present, which we term ‘convoluted’ (lower left), ‘folded’ (upper right), or ‘protruding’ (lower right, derived from another image of the same sample). A gallery of zoomed images of convoluted DNA structures is shown in Figure 2D. We imaged DNA samples of various CAG repeat lengths and analysed multiple images to produce a distribution of DNA forms for each sample. There was a progressive increase in the percentage of each type of unusual structure as the CAG repeat length increased, with over 50% of the molecules appearing unusual at the highest repeat length analysed (408 CAGs; Figure 2E).

The measured length of the linear molecules in the 360-repeat sample was 370±26 nm (n = 50), close to the expected 404 nm. It was not obvious from simple inspection whether the more complicated structures seen at longer repeat lengths consisted of single or multiple DNA molecules. To address this question, we measured the volumes of a number of linear molecules and a similar number of unusual structures in the sample containing DNA with 360 CAG repeats. The volumes determined (752±133 nm^3^ (n = 88) for the linear population and 802±158 nm^3^ (n = 72) for the unusual population) were not significantly different, indicating that the vast majority of the unusual structures consisted of single DNA molecules.

Adhesion of the DNA to the mica surface for the purpose of AFM imaging was routinely achieved using 10 mM MgCl_2_ in the adsorption buffer. To exclude the possibility that this concentration of Mg^2+^ was responsible for the generation of the unusual DNA structures in the DNA containing long CAG repeat lengths, in some experiments we coated the mica with poly-L-lysine before adsorption of the DNA in the absence of Mg^2+^. The positively charged poly-L-lysine coating binds DNA relatively strongly, so the DNA cannot equilibrate to its most energetically favourable two-dimensional conformation as it does in the presence of Mg^2+^. Consequently, we found that rather than being deposited in the relaxed conformation characteristically observed with the use of Mg^2+^ (Figure S1A), linear DNA (containing 360 CAG repeats) was deposited in serpentine conformations on poly-L-lysine (Figure S1B). However, this minor distorting tendency cannot account for the severe contortions observed in the figure: structures in which the DNA strand could not be traced from end to end were prevalent. Evidently, CAG repeat-induced conformational abnormalities do not depend on the presence of Mg^2+^.

### The Unusual Structures do not Include Extensive Regions of ssDNA

Given the repetitive sequence involved in these experiments, PCR synthesis in one direction might have been less effective than in the other, resulting in the generation of ssDNA. Other AFM studies (e.g. [35]) have shown that under some conditions ssDNA forms convoluted structures visibly distinguishable from dsDNA, which is characteristically linear and uncontorted. We therefore tested whether the longer repeat DNA molecules contained extensive regions of ssDNA. To do this, we used the restriction enzyme TseI, which cuts at the sequence 5′-GC(A/T)GC-3′. To remove any intra-stand base pairing that would exist in ss CAG repeat DNA, incubations with the enzyme were carried out at 80°C; conveniently, TseI is stable at this temperature. In an initial experiment we deliberately generated ssDNA by amplifying a test sequence (the human σ-1 receptor sequence; [36]), using PCR with a 50-fold excess of the forward primer. This reaction generated both 690-bp dsDNA and the corresponding ssDNA. In AFM images, the two populations were clearly distinguishable (Figure 3A). The dsDNA looked normal and linear, and identical to dsDNA generated using the same template but with equal concentrations of the two primers (Figure S2A). In contrast, the ssDNA was highly compacted, likely as a result of intra-stand base pairing. After an extensive TseI digestion at 80°C, the ssDNA subpopulation was unaffected (arrows in Figure 3B), whereas the dsDNA was digested into fragments of various sizes, as expected given that the σ-1 receptor sequence contains five TseI sites. σ-1 receptor DNA that was exclusively double-stranded was also efficiently cleaved by TseI, but in this case there was no residual ssDNA (Figure S2B). These results demonstrate that TseI cuts dsDNA that contains the appropriate target sequence, but not the corresponding ssDNA.

An AFM image of DNA containing 585 CAG repeats showed the usual pattern of normal and unusual structures (Figure 3C). An extensive digestion of this sample with TseI at 80°C resulted in the removal of all conformations of DNA (Figure 3D), showing that it was predominantly double stranded. A dense homogenous ‘background’ of short (∼6-bp) fragments remained, together with a few aggregates. One fragment of ∼60 bp, which likely represents a flanking region, can also be seen. Interestingly, when a limited TseI digestion was carried out at room temperature, the enzyme preferentially cleaved linear regions of DNA, leaving behind the contorted regions (Figure 3E, F). These results rule out the presence of extensive regions of ssDNA in the unusual structures, and also indicate that the convolutions sterically hinder access of the restriction enzyme to the DNA.

### The Unusual DNA Structures Include Hairpin Loops

**Figure 4:**
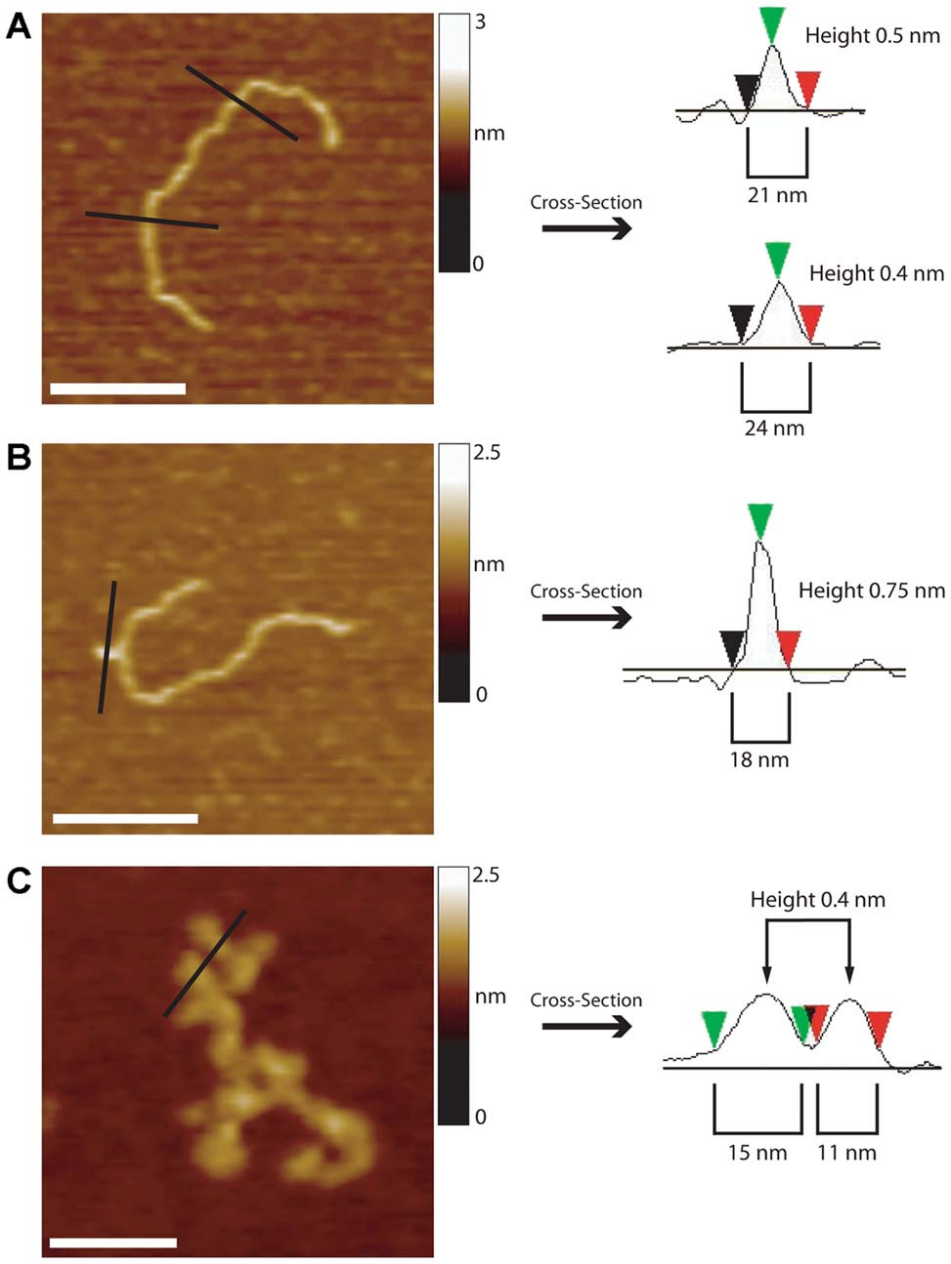
Sections through DNA molecules showing either normal or unusual structures. (A) Normal DNA (216 CAG repeats). Scale bar, 100 nm. (B) Protruding DNA (360 CAG repeats). The cross-section shows a width comparable to that of normal dsDNA but a greater height, suggesting an imperfect hairpin or hairpin-like structure. Scale bar, 100 nm. (C) Convoluted DNA (585 CAG repeats). The cross-section indicates protrusions similar in height and width to dsDNA. Scale bars, 50 nm.

To probe the nature of the unusual DNA structures further, we took cross-sections through them and compared the profiles to those of ‘normal’ DNA. A section through linear DNA indicates a height of 0.4 nm, which is typical of the height of double-stranded (ds) DNA, as determined by AFM imaging (e.g. [26]; Figure 4A). (The width of the DNA is considerably overestimated because of the geometry of the scanning tip, so this parameter does not yield useful information). By contrast, the height of a typical ‘protrusion’ (0.7 nm) is almost twice that of normal dsDNA (Figure 4B). This structure might therefore represent self-associating hairpins. The heights of the spurs in the convoluted DNA (Figure 4C) are close to that of normal dsDNA, suggesting that these structures might be single hairpins.

**Figure 5:**
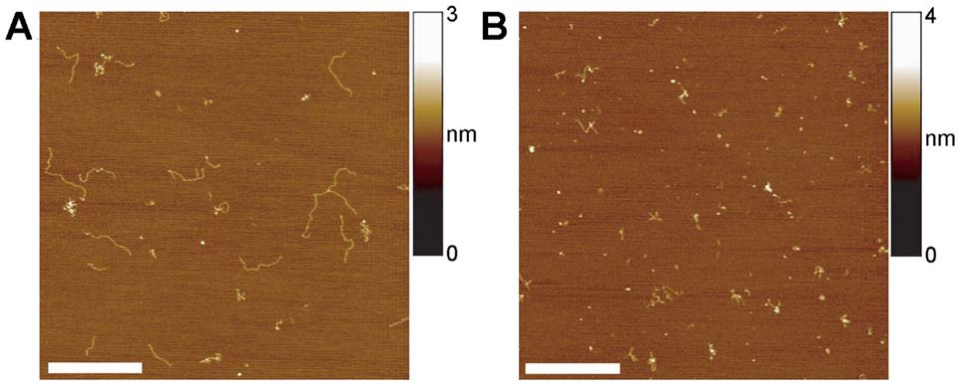
Effect of mung bean nuclease indicates the presence of hairpins in the convoluted molecules. (A) DNA containing 360 CAG repeats that had been treated with mung bean nuclease. Note the presence of an unusual number of short fragments. (B) The same DNA that had been treated with mung bean nuclease, and then melted and reannealed. Note the almost complete absence of full-length linear DNA. Scale bars, 250 nm.

If hairpin loops do exist within the convoluted DNA, then they will contain both short single-stranded regions at their tips, and mismatched bases. Both of these features should be susceptible to cleavage by mung bean nuclease [21], [22]. When DNA containing 360 CAG repeats was incubated with mung bean nuclease without subsequent melting, it appeared similar to undigested DNA, although there was an increase in the number of short fragments (Figure 5A). However, if the DNA was melted after the reaction and then allowed to reanneal, very little full-length linear DNA remained, and instead many smaller fragments were seen (Figure 5B). These data best fit a scenario in which mung bean nuclease cleaves mismatches in hairpins - generating some short fragments before melting - and also short ssDNA in hairpin loops, resulting first in nicks, and then in many very short fragments upon DNA melting. These results indicate that hairpin loops are indeed present in the convoluted DNA, and that even apparently ‘normal’ linear DNA contains short hairpins below the resolution limit of AFM.

### The Unusual Structures are Sensitive to Cleavage by the Type-III Restriction-Modification Enzyme EcoP15I

**Figure 6:**
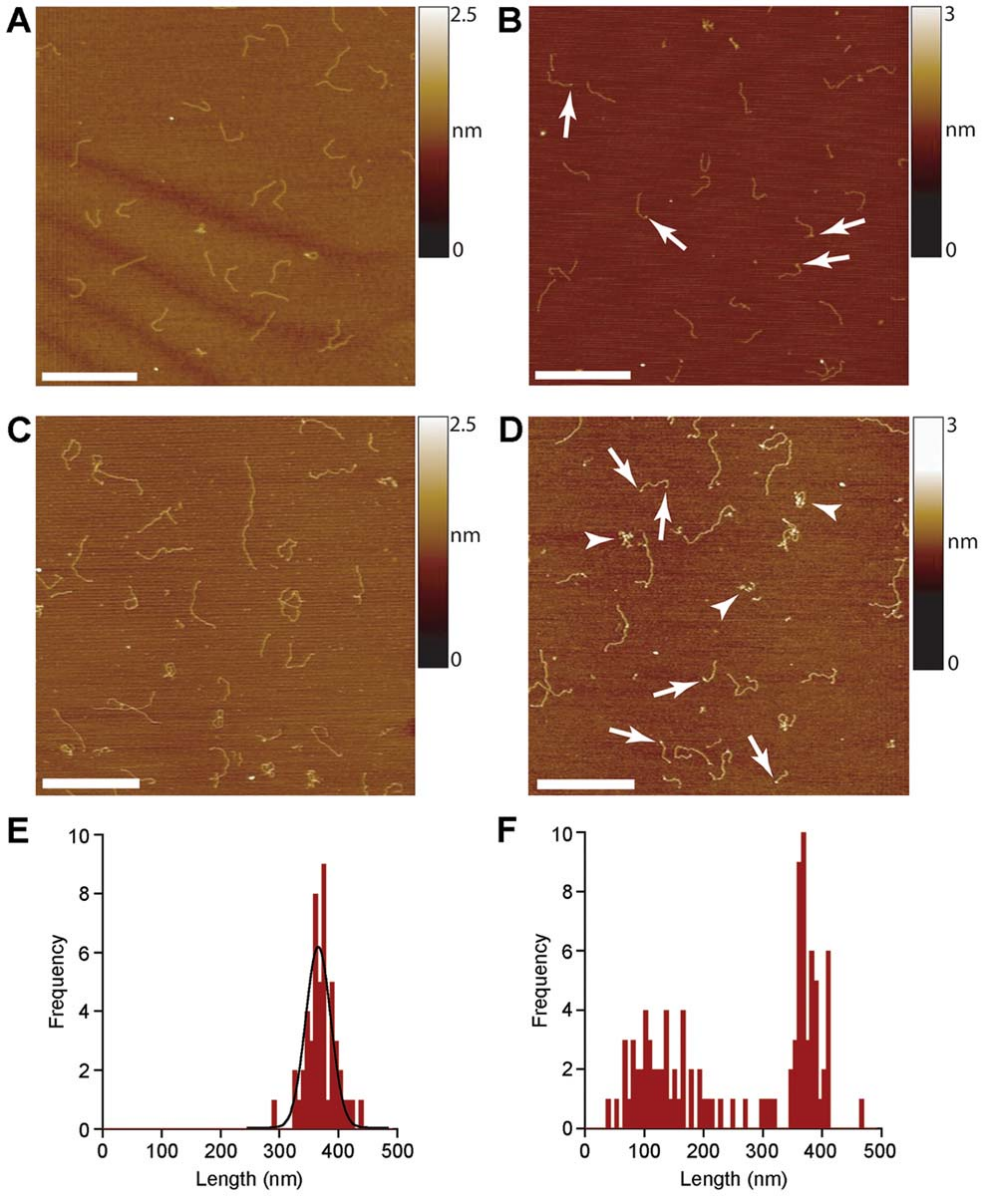
Convoluted DNA is selectively digested by EcoP15I. (A, B) AFM images of DNA with 216 CAG repeats (total length 740 bp) before (A) and after (B) incubation with EcoP15I. (C, D) DNA with 360 CAG repeats (total length 1211 bp) before (C) and after (D) incubation with EcoP15I. Arrows in (D) indicate DNA molecules bearing enzyme bound at the ends. Arrowheads indicate residual tightly-wound tangles. All scale bars, 500 nm. (E, F) Frequency distributions of lengths of linear DNA in untreated (E) and EcoP15I-treated (F) samples of 360-repeat DNA.

The sequence 5′-CAGCAG-3′ is the recognition sequence for the Type III restriction-modification enzyme EcoP15I [37], [38]. For efficient cleavage, EcoP15I normally requires the interaction of two enzyme complexes that bind to sites oriented in a head-to-head fashion. One enzyme translocates towards the other, bound at its target site, and cleavage occurs upon collision. It has been shown previously that EcoP15I cuts CAG repeat DNA, provided that a reverse recognition site is present in the plasmid [39], and it was demonstrated that this enzyme could be used to count the number of CAG repeats in a DNA sequence. We decided to test whether EcoP15I would selectively cut convoluted DNA, even though the DNA does not contain a reverse recognition sequence, reasoning that the geometry of the convolutions might allow collision between two enzyme complexes even in the absence of a reverse recognition sequence. We found that EcoP15I treatment did not cleave linear DNA containing a relatively short (216) CAG repeat (compare Figure 6A with Figure 6B). Interestingly, the treated molecules often bore blobs (arrows in Figure 6B), which are likely to be enzyme complexes that have stuck to the ends of the DNA. In contrast, EcoP15I treatment of DNA containing 360 CAG repeats led to the generation of shorter linear fragments (compare Figure 6C with Figure 6D), many of which again bore blobs at their ends (arrows in Figure 6D), small, tangled structures (arrowheads in Figure 6D), and normal full-length linear DNA. The frequency distribution of lengths for the linear subpopulation in the untreated sample (Figure 6E) shows a single peak, and the mean length was 370±26 nm (n = 50), as reported above. The corresponding distribution for linear DNA in the EcoP15I-digested sample (Figure 6F) has two peaks, one at 372±29 nm, and a broader peak, encompassing fragments with blobs at their ends, at 141±68 nm (n = 50). EcoP15I treatment also caused a fall in the volume of the convoluted structures from 910±330 nm^3^ to 380±190 nm^3^. These results indicate that EcoP15I does indeed preferentially digest the convoluted DNA, leaving behind a mixture of short linear fragments of various lengths and tightly-wound tangles.

### The Unusual Structures are Very Resistant to Unfolding

**Figure 7:**
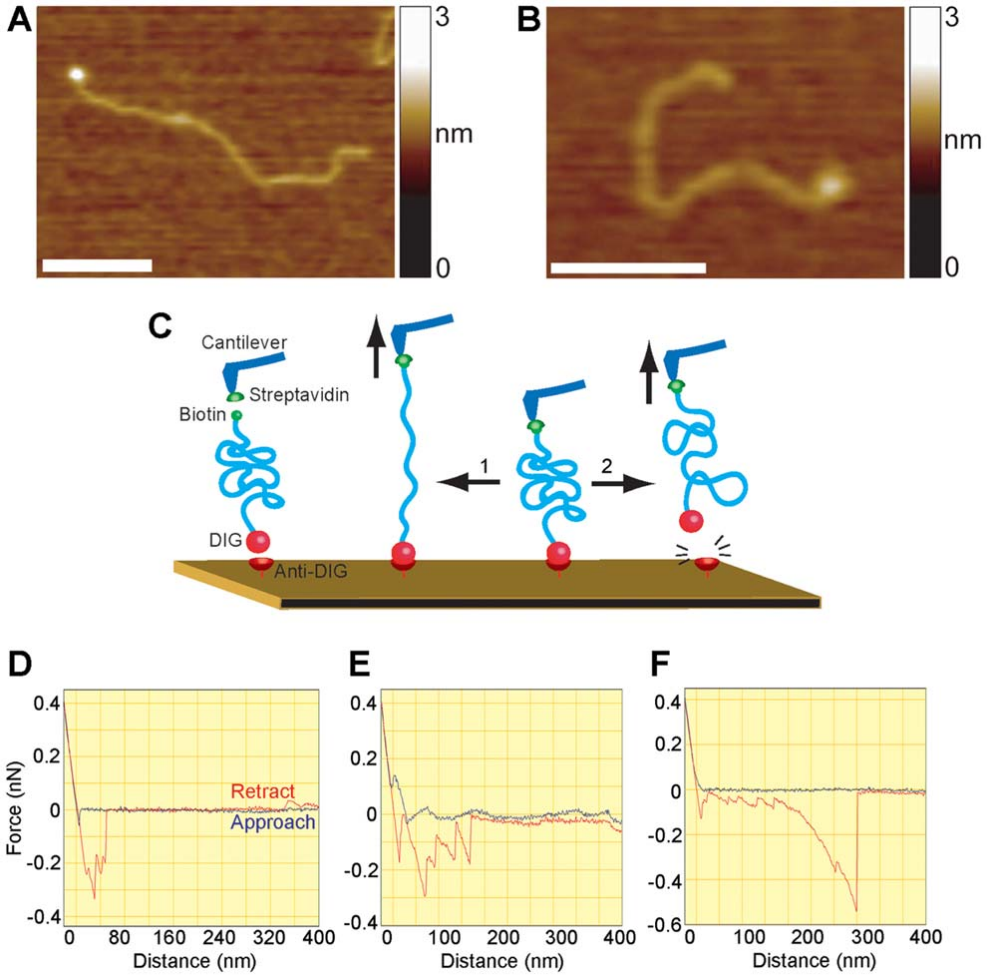
Convoluted structures resist considerable pulling force. (A) AFM image of a DNA molecule containing 360 CAG repeats (total length 1211 bp) showing a streptavidin molecule, attached to a terminal biotin moiety. Scale bar, 100 nm. (B) AFM image of a 272-repeat DNA molecule showing an anti-DIG antibody molecule, attached to a terminal DIG moiety. Scale bar, 100 nm. (C) Experimental rationale. A silicon nitride cantilever and a mica disk were both amino-functionalized by incubation with APTES. Glutaraldehyde was then used to couple streptavidin to the cantilever and anti-DIG to the mica. DNA fragments were bound to the steptavidin via a biotin tag on the DNA. The DNA was lowered onto the antibody-coated mica to allow the DIG tag on the DNA to bind to its antibody on the mica. The cantilever was then retracted, and the forces applied during the retraction are measured. Two possible outcomes are depicted: (1) the convoluted DNA is unwound; (2) the DNA remains convoluted and the antigen-antibody bond is ruptured. (D) Typical force curve for a DNA fragment containing 114 CAG repeats, showing ‘approach’ (blue) and ‘retract’ (red) traces. Note the multiple force peaks, the final rupture distance (41 nm) and force (200 pN). 14/150 retractions (9%) resulted in a measurable force peak. (E) Typical force curve for a DNA fragment containing 360 CAG repeats. Note the multiple force peaks, the final rupture distance (117 nm) and force (159 pN). 22/124 retractions (18%) resulted in a measurable force peak. (F) Force curve for a 360-repeat fragment showing a very long rupture distance (260 nm) and a large force (529 pN).

The results presented so far suggest that the convoluted DNA is not loosely coiled, but rather contains non-B-form structures. To test this idea further, we attempted to measure the force required to ‘untangle’ the convoluted DNA. We reasoned that loosely coiled DNA should be untangled at a relatively low force, but that hairpin loops, for example, should resist large pulling forces. We produced DNA containing either 114 or 360 CAG repeats, each bearing a biotin tag at one end (where the forward primer anneals) and a digoxigenin (DIG) tag at the other end (where the reverse primer anneals). AFM images showed that the biotin tag could be decorated with a streptavidin molecule (Figure 7A), and the DIG tag could be decorated with an anti-DIG antibody (Figure 7B).

For single-molecule force spectroscopy, the AFM cantilever was derivatized with streptavidin, and the mica surface was coated with covalently bound anti-DIG antibody (Figure 7C). DNA was attached to the streptavidin-derivatized cantilever via the biotin tag. The cantilever was then lowered onto the antibody-coated surface, retracted, and the pulling forces between the cantilever and the surface measured. A typical pulling trace for the 114-repeat DNA had multiple force peaks, a final rupture force of 200 pN, and a pulling distance before rupture of 41 nm (Figure 7D). With the 360-repeat DNA, multiple force peaks were again seen, and in the case shown, the final rupture force was 159 pN, and the pulling distance before rupture was 117 nm (Figure 7E). The mean pulling distance before rupture was greater for the long-repeat DNA (72±39 nm; S.D.; n = 22) than for the short-repeat DNA (32±15 nm; n = 14; P<0.01). The mean number of peaks over a series of pulling events was 2.00±0.75 for the short-repeat DNA and 2.82±0.85 for the long-repeat DNA. The mean final rupture forces were very similar for the two types of DNA (126±78 pN for the short-repeat DNA, and 125±55 pN for the long-repeat DNA).

Since both types of DNA had similar numbers of peaks per pulling event, despite the fact that only the long-repeat DNA has a significant number of convolutions, the multiple peaks likely represent the rupture of several DNA molecules conjugated at different points on the cantilever. The fact that the rupture forces are the same for the two types of DNA indicates that they represent the breakage of the same bond. This is likely to be the DIG-anti-DIG interaction, since antigen-antibody bonds have lower rupture forces (e.g. [40]) than the biotin-streptavidin bond (e.g. [41]). The fact that the rupture distance for the long-repeat DNA (72 nm) is much shorter than the total length of the DNA (370 nm) suggests that convoluted DNA is being attached to the cantilever. The distance across a typical convoluted molecule is about 100 nm (see Figure 2C, for example). Although the long-repeat DNA typically showed a rupture distance of less than 100 nm, in a few cases, a very long pulling distance was detected. In the example shown (Figure 7F), the pulling distance was 260 nm and the pulling force was 529 pN. These long pulls likely represent the behaviour of unconvoluted DNA present in the long-repeat sample. Overall, these pulling experiments demonstrate that the antigen-antibody bond is ruptured before the DNA convolutions are unwound, supporting the idea that the convoluted DNA contains stable structures such as hairpin loops.

## Discussion

AFM allows high-resolution, single-molecule imaging of DNA with minimal sample preparation. We have shown previously that this imaging method provides structural information not attainable using other methods, such as EM. In this study we have used AFM imaging of DNA containing super-long CAG repeats to reveal the presence of various unusual non-B-form structures, including hairpin loops and large convoluted structures. By contrast, in previous EM-based studies, the observed features consisted almost exclusively of short spurs and kinks [22], [23]. The unusual structures reported here are consistent with both the prediction [42] and experimental evidence [43] that DNA composed of long stretches of CAG repeats is particularly flexible. Importantly, we also show that the prevalence of these anomalous structures increases with increasing CAG repeat length. Maintained sensitivity to the restriction enzyme TseI rules out the presence of extensive regions of single-stranded DNA. On the other hand, sensitivity to digestion by mung bean nuclease is consistent with the presence of hairpin loops. The fact that forces sufficient to rupture antigen-antibody bonds fail to unwind the convoluted DNA indicates the presence of stable unusual DNA structures.

It is known that a significant change in the phenotype of the R6/2 mouse occurs at a repeat length of around 300 CAGs [17], [18]. Specifically, increases in repeat length have been shown to cause a delayed onset of disease and prolonged survival, from around 4 months to over 18 months in mice with the longest repeats [17]. Increased repeat length was associated with a reduced nuclear entry of the larger htt fragments [18], which will cause an amelioration of the R6/2 phenotype relating to the formation of nuclear inclusions [44], [45], [46]. A reduced transcription of the transgene was also seen at long repeat lengths, which is known to be associated with an amelioration of the R6/2 phenotype [16], [47]. We speculate that the generation of unusual DNA structures seen in our *in vitro* experiments might underlie the observed reduction in transcription observed *in vivo*. Significantly, it has been shown previously that Hdh mRNA levels are reduced in knock-in mice containing 150 CAG repeats [19]. Consistent with this observation, we saw fewer of the unusual structures in short repeat length DNA, but they were still present. It is not clear if there is a reduced level of mutant htt in mice with superlong CAG repeat lengths. We know that htt with superlong CAG repeats is translated, because bands of the appropriate size can be visualized using anti-htt antibodies when we run proteins from the allelic series of mice on immunoblots (data not shown). However, because mutant htt forms aggregates, accurate protein quantitation is not possible (for detailed discussion of this issue, see [17]).

In addition to the insights provided into the HD phenotype, our study also sheds light on the mechanism of action of two very different restriction enzymes. The observation that at 80°C Tse1 cleaves within the hairpins indicates that an A:A or T:T mismatch at the centre of the recognition sequence (5′-GC(A/T)GC-3′) does not prevent its restrictive activity. Tse1 may therefore belong to an interesting group of restriction enzymes which flip out nucleotides within a recognition sequence (e.g. [48]). In the few studied cases, the restriction enzyme uses a flipping mechanism to recognize a sequence or adjust the shape of the DNA within its catalytic site for proper cleavage. Either of these scenarios might occur when Tse1 encounters dsDNA. We suggest that nucleotide flipping may allow it to recognize and cleave near an A:A or T:T mismatch, or to collapse the recognition sequence to 5′-GCGC-3′, thereby ignoring mismatches. These possibilities remain to be tested.

The ability of EcoP15I to cleave the convoluted structures selectively is a particularly interesting phenomenon, given that two head-to-head recognition sites are normally required for its restriction activity [37], [38], [49]. Our data suggest that the EcoP15I enzyme may be recognizing the sequence 5′-CXGCXG-3′ where X represents the A:A or T:T mismatch. Significantly, a previous study of EcoP15I activity using 2-aminopurine substitutions within the cognate sequence [50] revealed distortion (probably base-flipping) at the two adenine sites upon enzyme binding. The same study also showed preferential binding to target sequences containing a mismatch. Preceding each hairpin will be perfect repeats containing the normal EcoP15I target sequence, 5′-CAGCAG-3′, which it would appear can interact with the mismatch sequence to produce cleavage. Further investigation of the action of these restriction enzymes on CAG-repeat DNA is clearly warranted.

With respect to the results presented here, the central question is: could the generation of unusual DNA structures play a role in the aetiology of HD? It is certainly clear that CAG repeats in the appropriate range exist in HD patients [9]. It is also known that the production of hairpin loops in triplet repeat DNA is a potential mechanism underlying repeat instability, which is a feature of triplet repeat disorders such as HD [2], [20], [51], [52]. It is not known, however, if abnormal secondary structures exist in DNA *in vivo*. The DNA imaged in our study was generated by PCR, which of course uses thermal cycling to drive melting and reannealing. However, a similar cycling between double- and single-stranded DNA states occurs during the processes of transcription, replication and recombination, suggesting that unusual DNA structures might also be generated *in vivo*. The observation that mRNA levels are reduced in HD mouse models with super-long CAG repeats supports this suggestion.

While CAG repeat disorders such as HD typically have a relatively short (<80) CAG repeat that causes disease, there are several studies showing that CAG repeat instability can generate CAG repeat lengths of greater than 300 in individual cells [10], [13]. There are also a large number of diseases, including myotonic dystrophy type 1 and Fragile X syndrome, where the disease-causing mutations consist of repeats (in non-coding regions of the genome) that are well above the size of repeats found here to generate unusual DNA structures. We speculate that if the conformational changes in DNA that we have described occur *in vivo*, they may contribute to pathology associated with trinucleotide repeat diseases. They could do this by allowing a gain of function, for example, by permitting cleavage by enzymes that normally do not cut, or by preventing binding of regulatory proteins to dysregulate transcription. They may also contribute to alterations in chromatin structure that are known to occur in CAG repeat diseases [53]. It is unlikely that the reduction of transcription causes the disease phenotype, given that only one functional allele of *HTT* is needed and that mutant *HTT* can rescue the lethality in null mutants. But it is also possible that the presence of super-long CAG repeat expansions may have a paradoxically ‘protective’ effect. For instance, by reducing transcription of the mutant DNA, they may reduce the amount of toxic mutant protein, and delay the onset of disease. However, these ideas must be regarded as speculative, since at present it is not possible to determine whether or not the abnormal DNA structures that we describe exist *in vivo*.

## Supporting Information

Figure S1 **Observation of convoluted DNA structures does not depend on the presence of Mg^2+^ in the deposition buffer.** DNA with 360 CAG repeats (total length 1211 bp) was deposited on mica that had been treated with either Mg^2+^ (A) or poly-L-lysine (B). Despite the absence of Mg^2+^ ions, the DNA bound via poly-L-lysine still exhibited anomalous, non-linear conformations. Scale bars, 500 nm.
(TIF)

Figure S2 **Human σ-1 receptor DNA shows no unusual structures.** (A) AFM image of undigested σ-1 receptor DNA. Note that the DNA is normal and linear. Scale bar, 250 nm. (B) σ-1receptor after complete Tse1 digestion at 80°C. Fragments of various sizes are visible after digestion, as expected since the σ-1 receptor sequence contains five TseI cleavage sites. Scale bar, 500 nm.
(TIF)
