# Supplementary figures and images for: Unusual Structures Are Present in DNA Fragments Containing Super-Long Huntingtin CAG Repeats
Source: PLoS One. 2011 Feb 11;6(2):e17119. doi: 10.1371/journal.pone.0017119 (PMC3037965; doi:10.1371/journal.pone.0017119)

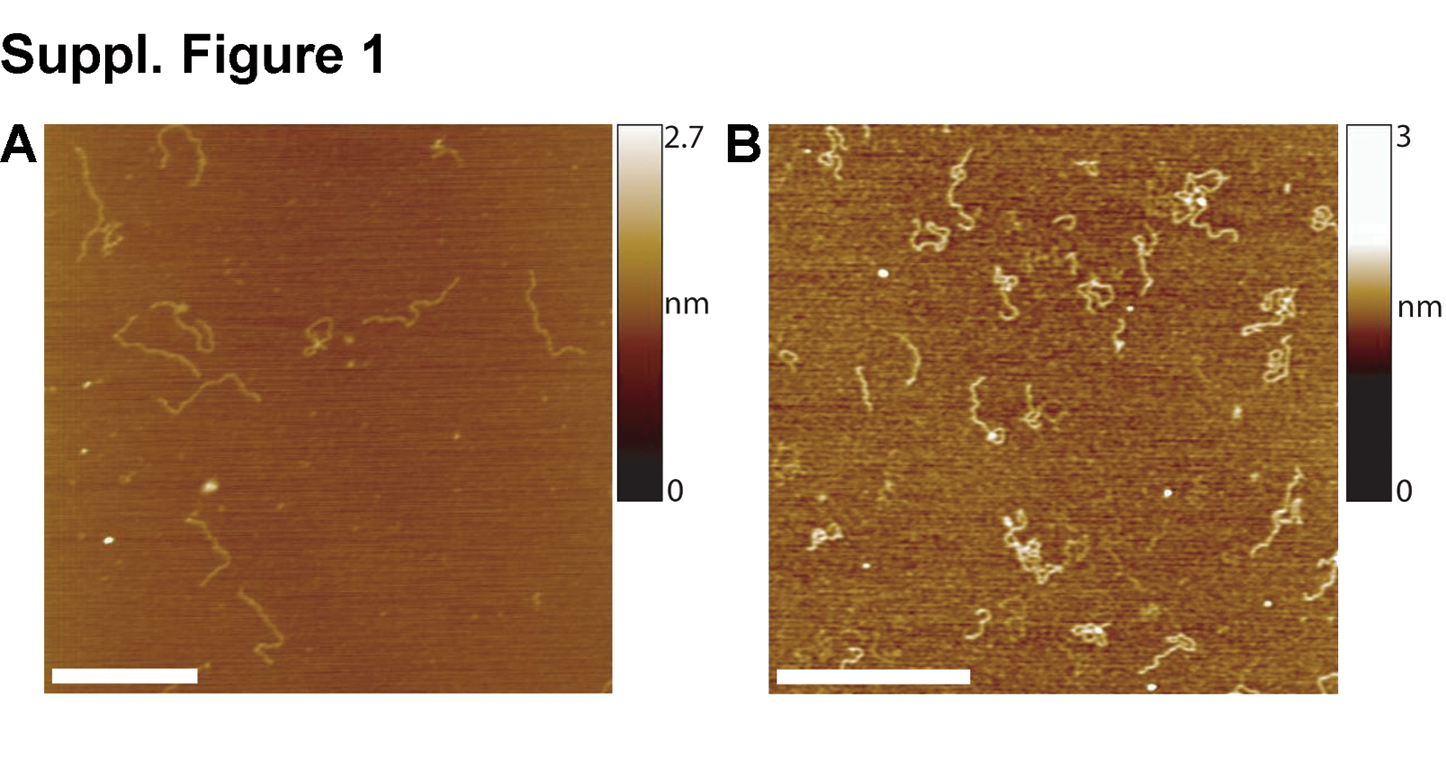

Supplement: Figure S1 — Observation of convoluted DNA structures does not depend on the presence of Mg2+ in the deposition buffer. DNA with 360 CAG repeats (total length 1211 bp) was deposited on mica that had been treated with either Mg2+ (A) or poly-L-lysine (B). Despite the absence of Mg2+ ions, the DNA bound via poly-L-lysine still exhibited anomalous, non-linear conformations. Scale bars, 500 nm. (TIF) [file pone.0017119.s001.tif]

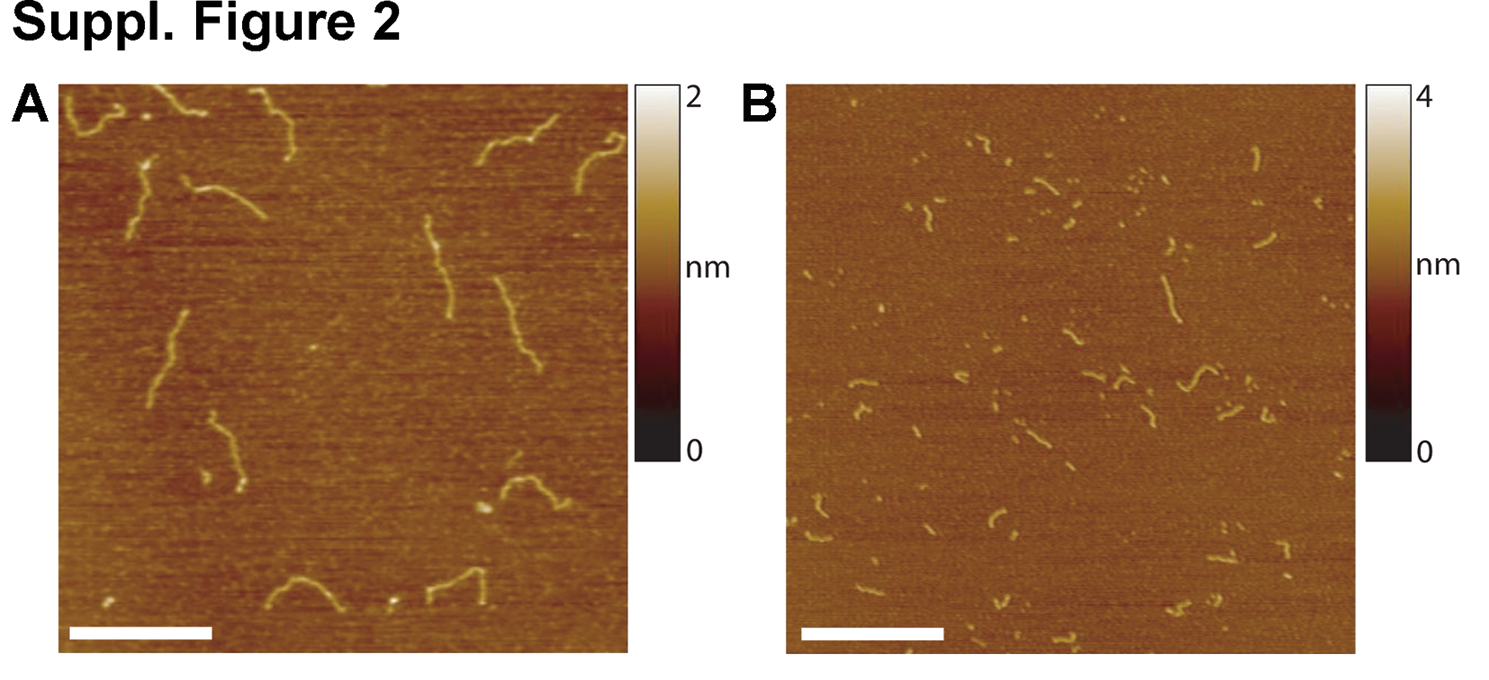

Supplement: Figure S2 — Human σ-1 receptor DNA shows no unusual structures. (A) AFM image of undigested σ-1 receptor DNA. Note that the DNA is normal and linear. Scale bar, 250 nm. (B) σ-1receptor after complete Tse1 digestion at 80°C. Fragments of various sizes are visible after digestion, as expected since the σ-1 receptor sequence contains five TseI cleavage sites. Scale bar, 500 nm. (TIF) [file pone.0017119.s002.tif]
